# Evaluation of Various Biological Activities of the Aerial Parts of *Scrophularia frigida* Growing in Iran.

**Published:** 2017

**Authors:** Parina Asgharian, Fariba Heshmati Afshar, Solmaz Asnaashari, Farzaneh Lotfipour, Behzad Baradaran, Elmira zolali, Elhameh Nikkhah, Abbas Delazar

**Affiliations:** a *Drug Applied Research Center, Tabriz University of Medical Sciences, Tabriz, Iran. *; b *Faculty of Pharmacy, Tabriz University of Medical Sciences, Tabriz, Iran. *; c *Student Research Committee, Faculty of Pharmacy, Tabriz University of Medical Sciences, Tabriz, Iran.*; d *Immunology Research Center, Tabriz University of Medical Sciences, Tabriz, Iran.*

**Keywords:** *Scrophularia frigida*, Antioxidant activity, Cytotoxic effect, Phytochemical analysis, NMR, GC-MS

## Abstract

The current study was assigned to evaluate the total phenol, total flavonoid content (TPC, TFC) and antioxidant properties of extracts from the aerial parts of *Scrophularia frigida* (*S. frigida*). Extracts were also tested by preliminary phytochemical screening as well as cytotoxic activity against *Artemia salina*, MCF-7 (human breast carcinoma) and SW-480 (colon carcinoma) and L-929 (normal) cell lines along with antimicrobial characteristic. DPPH, MTT and Brine shrimp lethality tests and disc diffusion method were carried out to determine the biological activities of the different extracts of *S. frigida*. In addition, the extracts which had more potent antioxidant and antiproliferative activity were further analyzed by NMR and GC-MS. 40% methanol-water (from MeOH extract) fraction showed higher amounts of TPC, TFC and antioxidant property. Findings of the study for general toxicity effect showed that dichloromethane (DCM) and MeOH extracts had weak to moderate effects. Furthermore, DCM extract indicated the most potent anti-proliferative activity against cancer cell lines. No evidence of antibacterial activity was determined. On the other hand, analysis of the potent extract DCM in cytotoxic assay showed the presence of *trans-*phytol and *cis*-oleic acid in GC-MS. Furthermore, NMR analysis of potent methanolic fractions in antioxidant tests revealed the presence of iridoids and phenolics. Generally, the results of TPC, TFC and antioxidant activity of extracts and fractions were in agreement with each other.

## Introduction

The genus *Scrophularia* L. from the Scrophulariaceae family, commonly known by the Persian name of «*gole meimuni»* and the English name «fig wort «or *«*brown –wort*»* is one of the most important and widely distributed genera in central Europe, Asia and North America, especially in the Mediterranean area (1). This genus is comprised of approximately 300 species worldwide, and 42 species are represented in Iranian flora, of which 19 species are endemic (2, 3). From ancient times, many species of this family have been used in traditional medicine for a variety of ailments such as skin disorders, inflammatory conditions and phytotherapy (3, 4). Existence of some classes of secondary metabolites such as iridoids and iridoid glycosides glycosides, phenyl propanoides, phenolic acids, flavonoids, saponins, and terpenoids were identified in many species of *Scrophularia,* which are correspond to biological activities including anti-inflammatory, anti-bacterial, cardiovascular, diuretic, protozoacidal, fungicidal, cytotoxic, anti-nociceptive and wound healing (5-15). *S. frigida* from the *Scrophulariaceae* family is a perennial herb with erect glabrous square stems of up to 80 cm in height. Leaves are opposite but in rare cases the upper leaves are alternating, glabrous and ovate or ovate-lanceolate, the highest of which angust linear, has inflorescence that is cyme-raceme with open two-lipped flowers called *S. persica* (local name: gole meimuniye yakhchali) and is endemic in different regions of Iran (16). To the best of our knowledge, no study has yet been done on this species. Therefore, as part of our ongoing phytochemical and bioactivity studies on Iranian plants (17, 18), we have now conducted to evaluate the total phenolic content (TPC), total flavonoid content (TFC) and antioxidant activity (I), as well as general toxicity and cytotoxic activities (II) along with anti-bacterial activity of extracts obtained from the aerial parts of the above-mentioned plant (III) by relative test as well as making comparisons of biological activities recorded in a previous investigation (IV). Moreover, the preliminary phytochemical analyses were evaluated to determine the presence of secondary metabolites (V). Finally, the active extracts were further subjected to GC-MS and NMR analysis for identifying of the active compounds present in the extract (VI).

## Material and method

*Chemicals*

3(4,5-dimethylthiazol-2-yl)2,5-diphenyl-tetrazolium bromide (MTT; Sigma-Aldrich, USA); 2,2-diphenyl-1-picrylhydrazyl (DPPH), rutin, gallic acid, Folin-Ciocalteu reagent, aluminum chloride, penicillin G-streptomycin and fetal bovine serum (FBS), all from Sigma -Aldrich chemical company (Germany), RPMI 1640 from Gibco, UK, phosphate buffer saline (PBS), Muller Hinton Agar Medium (MERCK) and Trypsin–EDTA (Gibco, Paisley, UK) were purchased. All other reagents and chemicals were of analytical grade. Solvents used for extraction and tests from Caledon and Scharlau.

*Plant Material*

The aerial parts of *S. frigida* were collected at the flowering stage from Mishodagh mountain which located in Yam, near the Marand city, at E: 45 48 03, N: 38 19 36 (altitude of 1999) in East Azarbaijan province (Iran) during August 2012. The identity of the plant was proved by anatomical and structural evaluation compared with the herbarium specimens. Voucher specimen under the accession code TBZ- fph- 746 representing this collection, has been maintained in the Herbarium of the Faculty of pharmacy, Tabriz University of Medical Sciences, Iran.

*Extract Preparation and Fractionation*

Air-dried and powdered aerial parts of *S. frigida* 100 g were subjected to soxhlet extractor to obtain n-hexane, dichloromethane (DCM) and methanol (MeOH) extracts, successively. In order to carry out more biological studies on MeOH extract fractions, solid-phase extraction (SPE) method was applied. 2 g of MeOH extract was loaded on a Sep-pak (10g:C18) cartridge (Waters, Ireland) and eluted by step gradients of methanol- water mixtures (10:90, 20:80, 40:60, 60:40, 80:20 and 100:0). Rotary evaporator (Heildolph, Schwabach, Germany) was used to remove solvents from different extracts and fractions at a maximum temperature of 45 °C and a very low pressure. The samples were stored in a freezer at -20 °C until further examinations.

*Antimicrobial assay*

The lyophilized form of microorganisms that were purchased from the Persian Type Culture Collection (Iran) included: 2 strains of gram negative species, *Pseudomonas* *aeruginosa *(ATCC 9027) and *Escherichia coli* (ATCC 8739), as well as the gram positive species namely *Staphylococcus epidermidis* (ATCC 12228) and *Staphylococcus aureus* (ATCC 6538), and a fungi (*Candida albicans*) (ATCC 10231) which were used to evaluate *in vitro* qualitative antimicrobial properties of the different extracts (n-hexane, DCM, MeOH) of *S. frigida*. Agar disc diffusion method was used for this aim. Activated bacteria were transferred in to the Muller Hinton Broth to incubate over night at 37 °C. Then, the centrifuged pellets (3000 rpm for 15 min) washed twice and re-suspended in Saline solution to provide an optical density equal to 0.5 McFarland (10 ^8 ^CFU/mL as a standard optical density). Then for providing the final concentration about 10 ^6 ^CFU/mL for inoculums, sterile Saline solution was used. In order to adjust the homogeneity of microbial growth, 10 mL of prepared inoculums suspensions were spread over the autoclaved Muller Hinton Agar Medium. Sterile discs (whatman paper no. 6 mm diameter) were placed on the surface of the media , were impregnated with 50 µL of different concentrations of extracts (1:1, 1:5, 1:10) which were dissolved in 50% aqueous DMSO. The plates were incubated for 30 min in a refrigerator to allow the diffusion of oil, and then they were incubated at 37 °C for 24 h. After this period, the inhibition zones obtained around sterile discs were measured (19, 20). In order to compare the potency of the anti-microbial activity of the extracts two control groups were considered: 1.aqueous DMSO as a vehicle control, 2. A standard disc of amikacin as a positive. All experiments were performed in duplicate and mean ± SD value was calculated.

*Anti-proliferative activity*

*Cell culture*

MCF-7 cells (human breast carcinoma cell line), L-929 (normal cell line) and SW-480 (colon carcinoma) were obtained from Pasture Institute, Tehran, Iran. All cell lines were maintained in RPMI 1640 as a cell culture medium with suitable additives containing 10% fetal bovine serum (FBS), 100 mg/mL streptomycin and 100 units/mL penicillin G. They were incubated in a humidified air/carbon dioxide (95:5) atmosphere at 37 °C. At 75% confluence, in order to rinsing and harvesting the cells from the flasks, phosphate buffered saline (PBS)/0.5% ethylenediamine tetraacetate (EDTA) and 0.25% trypsin/ EDTA solution were used respectively. Then sub-cultured them in to 96-well plates (Nunc, Denmark).

*MTT assay*

Microculture tetrazolium/formazan colorimetric assay was used for evaluating the anti-proliferative activity of medicinal plants (21, 22). For MTT assay 1×10^4 ^cells/well were seeded in to 96-well plates and incubated for 24 h allow to growing. Afterwards, for treating the cells, different dilutions (included: 1, 10, 100, 1000 µg/mL) of extracts (n-hexane, DCM and MeOH), which were prepared in dimethylsulfoxide (DMSO) and were diluted with cell culture medium, were added to cells and transferred to incubator. After 24 and 48 h of incubation for all cells the medium was replaced with a fresh medium containing (3-(4, 5-dimethylthiazol-2-yl)-2, 5-diphenyltetrazolium bromide) (MTT) reagent which this powder was dissolved in PBS to obtain 5 mg/mL solution. After a 4-hour incubation in air/carbon dioxide (95:5) atmosphere at 37 °C, the medium was removed and 100 µL of DMSO solvent was added to dissolve the formazan crystals completely. Microplate (ELISA plate reader, Bio Teck, Bad Friedrichshall, Germany) reader at absorbance of 570 nm for determining the formazan crystals (the metabolized MTT production) was used. Each experiment was performed in triplicate. For comparing the anti-proliferative activity of plants, Paclitaxel and DMSO were considered as positive and negative controls. The inhibitory rate was calculated by the following equation: 

Relative viability (%) = (A _test_/A _control_) ×100. 

Where A _control _is the absorbance of the control reaction (containing all reagents except the plant extracts) and A _test_ is the absorbance of the sample. IC_50_ value was defined as the concentration of the extract to produce a 50% reduction in viability of the cells relative to the negative control and calculated from a dose-response curve plotted in the Sigma Plot 10 software (23, 24).

*Assay for total phenolics content (TPC)*

Determination of total phenolic constituents of all extracts and fractions were evaluated using slight modified Folin - Ciocalteau`s test colorimetrically (25).

This method is based on the reducing power of Folin - ciocalteu reagent to produce blue color in the samples containing polyphenols. Briefly, 1 mL of prepared extracts and fractions (5 mg in aqueous acetone 60%) were mixed with 2 mL of Folin-ciocalteu reagent and 1 mL of aqueous Na_2_CO_3_. Afterwards, the complex mixture was centrifuged in 1200 rpm for 5 min. After incubation at room temperature for 30 min, absorbance of upper mixture was measured at 750 nm using UV spectrophotometer (Pharmacia Biotech Ultrospec 2000, UV/Visible Spectrophotometer, England) against negative control (reagent with no extracts and fractions). All the process mentioned above were applied for the different concentrations of gallic acid solution as a standard to prepare a calibration curve.TPC were expressed as gallic acid equivalent in mg per gram of dried extract. 

*Estimating the total flavonoid contents (TFC)*

Total flavonoid constituents of the extracts and fractions were assessed by involving the aluminium chloride reagent (consists of AlCl_3_ crystals plus sodium acetate crystals in 100 mL of 80% of methanol) and rutin as a standard, leading a modified colorimetric assay (26). Concisely, 2 mL of all samples (previously were dissolved in 80% methanol) were mixed with 400 µL of distilled water and 1 mL of AlCl_3_ reagent. Thereafter, mixtures were allowed to remain at room temperature for 30 min. Then the absorbance of the reaction mixtures was read at 430 nm vs blank spectrophotometrically. 5-25 µg/mL dilutions of rutin in 80% methanol were prepared in the same way and were used to calculate calibration curve in order to determining the quantitave flavonoid. Finally, TFC was expressed as rutin equivalents per gram of dried plant material.

*Brine shrimp lethality test (BSLT)*

Brine shrimp lethality assay was applied for evaluating the general toxicity of different extracts of *S. frigida*, through the estimation of the LC_50_ values which was described by slight modified Meyer and et al bioassay (27). Concisely, stock of each extract was prepared in DMSO (v/v) and diluted with artificial sea water so that final concentration of DMSO did not exceed 1%. Afterwards, serial dilution for obtaining different concentrations was used. Then *Artemia salina (A. salina*) (Sera, Turkey) eggs were hatched in a covered tank with artificial sea water under a bright light source for 48 h. When the eggs turn to nauplii larve, 10 of them were added in to each of test tubes and control (containing DMSO and sea water, no extract). After 24 h of incubation time, when the survival shrimps were faced with extracts, the number of survival larves was assessed. LC_50_ values were calculated from linear regression analysis and plotted concentration versus percentage lethality.

*Free radical scavenging activity test*

Antioxidant activity of the extracts was assessed spectrophotometrically using the 2, 2-diphenyl-1-picrylhydrazyl (DPPH) radical (molecular formula C_18_H_12_N_5_O_6_, molecular weight 394) obtained from Sigma -Aldrich Company. The DPPH assay was carried out as described by Takao *et al* (28). Stock solutions of extracts were prepared as 1 mg/mL in chloroform (CHCl_3_) for non-polar extracts and in MeOH for polar ones. 

Serial dilutions were made to obtain concentrations of 5×10^-1^, 2.5×10^-1^,1.25×10^- 1^, 6.25×10^-2^, 3.13×10^-2^ and 1.56×10^-2 ^mg/mL. Diluted solutions of extracts (5 mL each) were mixed with 0.08 mg/mL DPPH solution (5 mL) and allowed to stand for 30 min for occurring any reaction. The UV absorbance was recorded at 517 nm. The experiment was done in triplicate and the reduction of free radical DPPH in percent (R %) was calculated in the following way:

R% = (A _blank_ – A _sapmle_)/ A _blank_) × 100

Where A _blank_ is the absorbance of the negative control (containing all the reagents except the extract), and A _sapmle_ is the absorbance of the test samples. Extract concentration providing 50% reduction (RC_50_) was calculated from the graph plotting reduction percentage against extract concentration. Quercetine was used as positive control.

*Phytochemical analysis of different Crude extracts*

Extracts were tested to identify the active chemical groups such as triterpenoids, steroids, glycosides, saponins, alkaloids, flavonoids, tannins, free amino acids, iridoids and carbohydrate. Following standard procedures were used (29-32) 

*A: Tests for Steroids and Triterpenoids:*

Libermann-Buchard test: Few drops of acetic anhydride were added to the different extracts, then mixed. Color changes over a period of one hour were observed after addition of concentrated H_2_SO_4 _slowly, which was caused to formation of brown ring at the gap of two layers. Changes were as follows: appearance of bluish green color at the top layer is considered to presence of steroids and red color in the lower layer is an indicator of triterpenoids. 

*B: Tests for Glycosides*

After getting positive results from Libermann-Buchard tests, to determine the presence of unsaturated lactones and deoxy sugars, Kedd and keller killiani tests, which are characteristic of the cardiac glycosidecompounds, were done respectively.

*Kedd΄s test*

 2-3drops of 2%, 3, 5 dinitro benzoic acid (3, 5-dinitro benzene carboxylic acid -Keddeʹs reagent) in 90% alcohol were added to dry extracts, then the solution was set in alkali range with 20% KOH. In the presence of β-unsaturated -O- lactones, a purple color was observed.

*Keller-killiani test*

The mixture of glacial acetic acid and ferric chloride was added to dried test solutions. Detection of the color change to bluish green in upper layer and reddish in down layer was occurred after adding the concentration H_2_SO_4_ slowly by the side of the test tube.

*C: Tests for Alkaloids*

*Dragendorff’s test*

In the presence of potassium bismuth iodide solution as a Dragendorff reagent, reddish brown precipitate was appeared. 

*Hagerʹs test*

Saturated solution of picric acid as the Hager reagent was added to the test specimens. The presence of alkaloids leads to formation of yellow precipitate.

*D. Test for Tannins and phenolic compounds:*

Amount of 5% FeCl_3_ solution was added to tubes of extracts in the presence of tannins. Dark green color was appeared (33). 

*E. Test for flavonoids*

*Shinoda test:*

Drop wise of concentrated HCL with extract solutions was mixed, then one piece of Magnesium ribbon added, which was accelerated the speed of color changes to red. 

*F. Test for Amino Acids*

Ninhydrin test: Amino acids when boiled with 0.2% solution of Indane 1, 2, 3 trione hydrate, it gives violet after a few minutes.

*G. Test for Carbohydrate*

*Benedict’s test*

The mixture of test solution and drop wise of Benedict’s reagent, when boiled in water bath, were caused the formation of reddish brown precipitate, if carbohydrates are present.

*H. Tests for Iridoids*

1 mL of Trim-Hill reagent was added to the different extracts and then was heated for a few minute. A blue-green or red color indicated the presence of iridoids.

*GC-MS Analysis of potent Extract in anticancer bioassay*

GC–MS analyses were carried out on a Shimadzu QP-5050A GC–MS system equipped with a DB-1 fused silica column (60 m × 0.25 mm i.d., film thickness 0.25 μM).

For DCM extract oven temperature, rising from 50 °C to 230 °C at a rate of 4 °C/Min and then rising from 230 °C to 310 °C at a rate of 1.5 °C/Min, injector temperature, 280 °C; carrier gas, helium at a flow rate of 1.3 mL/min; split ratio, 1:10; ionization energy, 70 eV; scan time, 1 s; mass range, 30–600 amu.

*Identification of Components*

Identification of the constituents was based on direct comparison of the retention times and mass spectral data with those for standard compounds and computer matching with the NIST 21, NIST 107 and WILEY229 library, as well as by comparison of the fragmentation patterns of the mass spectra with those reported in the literature (34).

*NMR spectra from methanolic fractions*

NMR spectra were recorded in CD3OD and D_2 _O on a Bruker 200 MHz NMR spectrometer. TMS was used as the internal standard.

*Statistical Analysis*

All experiments were conducted in triplicate measurements and presented as the Mean ± SD. Data were analyzed by Excel 2010 Microsoft. The IC_50_ value was calculated from nonlinear regression analysis.

## Results

In the present study, antioxidant activity, TPC, TFC, general toxicity, cytotoxic effects and phytochemical analysis of three extracts with different polarity obtained from the aerial parts of *S. frigida* were determined and the results were shown in Table 1, 2 and 3.

*Antioxidant characteristic of the S. frigida extracts and its fractions*

The results of inhibiting free radicals obtained from extracts and Sep-pak fractions of *S. frigida* are given in Table 1. A simple and rapid assay to estimate free radical scavenging activity of the extracts and fractions is based on a color change, turning the purple color of unstable DPPH to the yellow color of stable DPPH-H by capturing hydrogen. In the current study, potent extracts and fractions showed antioxidant activities in a concentration-dependent manner. RC_50 _values of MeOH extract, 40% and 60% Sep-pak fractions (0.134 ± 0.04, 0.028 ± 0.00 and 0.035 ± 0.00 mg/mL respectively) in comparison with the RC_50 _value of quercetine as a positive control (0.003 ± 0.00 mg/mL) showed a moderate activities.

**Table1:** TPC, TFC and antioxidant activities of n-Hexane, dichloromethane and methanol extracts and sep-pak methanolic fractions of *S. frigida* aerial parts

**Extracts or fractions**	**Total phenol content (as gallic acid equivalents) mg g-1**	**Flavonoid content (mg g** ^-1^ **)**	**Antioxidant activity (RC50; mg mL** ^-1^ **)(a)**
MeOH	25.67 ± 0.07	72.08 ± 1.63	0.134 ± 0.04
DCM	37.89 ±2.18	96.60 ± 17.29	0.374 ± 0.08
n-hexane	4.79 ± 0.05	-	2.098 ± 1.63
10%	6.54 ± 0.10	9.05 ± 0.07	0.679 ± 0.10
20%	53.86 ± 0.79	24.86 ± 0.77	0.141 ± 0.05
40%	145.73 ± 1.81	101.28 ± 2.88	0.028 ± 0.00
60%	136.76 ± 0.26	99.85 ± 3.81	0.035 ± 0.00
80%	28.99 ± 0.47	73.68 ± 2.64	0.194 ± 0.02
100%	3.89 ± 0.23	15.60 ± 0.62	0.287 ± 0.13

Experiment was performed in triplicate and expressed as Mean ± SD.

** The RC_50_ value for quercetin as positive control was 0.003 ± 0.00 mg/mL.

**Table2:** Cytotoxicity (IC_50 _) of DCM and MeOH extracts of *S. frigida* aerial parts

**Samples**	**MTT assay**					
	**MCF7** **(g/mL)**		**SW480 ** **(g/mL)**		**L929 ** **(g/mL)**	
	**24 h**	**48h**	**24h**	**48h**	**24h**	**48h**
DCM	136.72 ± 15.45	0.97 ± 0.54	287.52 ± 30.36	4.11 ± 2.69	>1000	33.38 ± 1.25
MeOH	561.69 ± 46.71	202.77 ± 49.22	>1000	335.68 ± 56.28	>1000	138.49 ± 43.25

**Table 3:** Phytochemical tests for various extracts of *S. frigida* aerial parts

**Phytochemical tests**		**MeOH extract**	**DCM extract**	**n-hexane extract**
Alkaloids	Dragendorff’s Test	+	-	-
	Hager’s Test	+	-	-
Glycosides	Kedd	-	+++	+++
	Keller-killiani	-	-	-
Tannins	Ferric Chloride Test	+++	-	-
Flavonoids	Shinoda Test	+++	-	-
Proteins and Amino Acids	Ninhydrin Test	-	-	-
Sterol	Libermann-Buchard test	-	-	+
terpenoid	Libermann-Buchard test	-	+++	-
Carbohydrates	Benedict's test	+	-	-
Iridoids	Trim hill	+++	-	

**Table 4:** Composition of DCM extract of *S. frigida *aerial parts

**Extract**	**Total identified content (%)**	**Compound (content %)**
DCM	59.87	*Trans*-phytol
	26.91	*Cis*-oleic acid

*Total phenolic content*

The results of total phenolic constituents were quantified as standard gallic acid equivalents, using Folin- Ciocalteau`s method by reference to calibration curve. (y = 37.68x-0.0083, R^2 ^=0.9999). According to the results which were showed in Table 1. It was found that MeOH extract, 40% and 60% MeOH-Water Sep-pak fractions with (25.67 ± 0.07, 145.73 ± 1.81 and 136.76 ± 0.26 mg GAE/1 g of extract sample respectively) were superior to the phenolic content of other extracts and fractions.

*Total flavonoid content*

The total flavonoid contents of extracts and fractions were determined using AlCl_3 _reagent method and shown in Table 1. The amount of flavonoids in samples was calculated from the standard curve of rutoside as the standard flavonoid (y _= _110.29x + 4.1489, r^2 ^_= _0.9997). The results were represented as rutoside equivalents in mg g^-1 ^dry extracts and fractions. It is notable that the TFC content of MeOH extract, 40% and 60% Sep-pak fractions (72.08 ± 1.63, 101.28 ± 2.88 and 99.85 ± 3.81 mg rutoside equivalent in 1 g of powdered plant material, respectively) possesses the substantial amount of flavonoids than other extracts and fractions. 

*General toxicity*

Valid and rapid preliminary Brine shrimp lethality bioassay was utilized for comparing the cytotoxic activity of the extracts with positive control (Podophyllotoxin LD_50 _= 2.69 µg/mL). Results illustrated that the DCM and MeOH extracts with LD_50 _values of 71.55 ± 2.37 and 57.19 ± 9.67 µg/mL respectively, showed weak to moderate effect. In terms of n-hexane extracts no significant effect was observed.

*Cytotoxic activity*

Based on the BSLT results, the DCM and MeOH extracts of *S. frigida* showed acceptable effects in comparison to n-hexane extract. Therefore, these extracts were candidated for further cytotoxic investigations on the SW-480 and MCF-7 cell lines as the cancerous cells and L-929 as the normal cell line during 24 and 48 h period. Hence, MTT assay was carried out for evaluating the cytotoxic effects. IC_50_ values (necessary dose for 50% inhibition) for more clarifying the results were shown in Table 2. According to the results, the DCM extract revealed the most cytotoxic activity on both SW-480 and MCF-7 cell lines at 24h incubation. In addition, IC_50 _value of DCM extract on the MCF_-_7cell (136.72 ± 15.4 µg/mL) was lower than SW-480 cell line (287.52 ± 30.36 µg/mL). Whereas, the anti-proliferative activity of DCM extract against MCF-7 cell lines was remarkably significant in the first period (24 h), MeOH extract showed lower activity with IC_50 _ value of 561.69 ± 46.71 μg/mL. Moreover, as the time passed, DCM and MeOH extracts indicated more significant cytotoxicity in a time and dose dependent manner. This means that the IC_50 _value of DCM extract at second period incubation (48 h) was more decreased in comparison with the first period on MCF-7 and SW-480 cell lines with IC_50 _values of 0.97 ± 0.54 µg/mL and 4.11 ± 2.69 µg/mL, respectively. Similarly, more noticeable IC_50 _amount for MeOH extract on MCF-7 and SW-480 was achieved in comparison with the first period (24 h) (202.77 ± 49.22 µg/mL and 335.68 ± 56.28 µg/mL respectively). L-929 as a model of normal cell was not influenced by test specimens after 24 h (IC_50 _>1000 µg/mL). However; after the end of 48 h cytotoxic activity was observed by IC_50 _values of 33.38 ± 1.25 µg/mL and 138.49 ± 43.25 µg/mL for DCM and MeOH extracts, respectively. Our findings showed that the cytotoxic effect of DCM extract on non-cancerous cells is insignificant in comparison to its effect on cancer cells. This means that DCM extract are capable of selecting cancer cell line, while MeOH extract could not distinguish between normal and cancer cell types. Hence, it could be claimed that MeOH extract not only had no effect on cancer cells at the first period (24 h) but also it showed cytotoxic effect on normal cell at the end of the period (48 h).

*Antimicrobial activity*

No antibacterial activity of test specimens was observed against studied microorganisms.

*Phytochemical analysis*

Based on the results of preliminary phytochemical analysis which were shown in Table 3. MeOH extract consists of high amounts of flavonoids, tannins, carbohydrates, iridoids and alkaloids whereas in case of DCM and n-hexane extracts terpenoids and sterol were the major components, respectively. Additionally, glycosides were found in DCM and n-hexane extracts at almost the same level. 

*GC-MS results*

The results of GC-MS analysis of DCM extract of the aerial parts from *S. frigida* have been demonstrated in Table 4. *Trans*- phytol and oleic acid were in high amounts in DCM extract.

*NMR analysis*

NMR analysis were showed the presence of Iridoids and phenolic compounds in different fractions. 

## Discussion

In the present study BSLT, which is a valid, cheap and available method to test the aerial parts of specimens, was used as the first step for evaluating cytotoxic effects (35). The results suggested that MeOH extract of *S. frigida* was effective against *A. salina* in comparison to other extracts. Furthermore, DCM extract indicated weak toxicity against *A. salina*, while n-hexane extract had no effect. In the next step, for further assessment of cytotoxic activity of MeOH and DCM extracts, MTT assay was applied on 2 cancer and 1 normal cell lines. According to the results, extracts demonstrated different effects on cell lines in a dose and time dependent manner. DCM extract indicated a high and selective anti-proliferative effect on the studied cells. The potent activity of DCM extract in comparison to others might be due to the existence of high amounts of cytotoxic compounds in this extract. Additionally, selectivity of DCM extract to cancerous cells without significant damage to non- cancerous cells encourages consideration of DCM extract for further *in-vivo* studies. These findings are in agreement with several other studies in the field that describe the cytotoxic role of some species of *Scrophularia* on cancerous cells with different mechanisms. As an illustration, the cytotoxic activity of extracts from aerial parts of *S. oxysepala* in breast cancer cells via inhibition of cell growth and induction of apoptosis was approved in a recent study (36). Furthermore, presence of some phenolic acids in *Scrophularia* species, which have shown cytotoxic and cytostatic activities vs various cancer cell lines, were responsible for the chemo preventive effect of this genus (37). These results are consistent with the custom of popular folk medicine of using some species of *Scrophularia* to treat cancer (38). According to the instructions of the National Cancer Institute (NCI) it is valuable to invest crude extracts possessing an IC_50 _at a dose less than 20 µg/mL. After this screening step more analyses should be run to obtain pure compounds and to determine their structures (39). Based on this criterion (benchmark), the DCM extract with IC_50 _< 20 µg/mL on MCF-7 and SW-480 cell types can be considered as a candidate for further analysis and future research. In summary, based on these findings, the DCM extract can be considered as a successful agent and serve to demonstrate its novel potential as a natural source of anti-cancer drug. Subsequently, the observed cytotoxic properties of DCM extract have led us to analyze it and identify some active functional groups. GC-MS analysis of DCM extract and preliminary phytochemical studies of extracts revealed presence of different groups of organic compounds in the extracts.

In previous investigations, the cytotoxic effects of natural compounds such as various types of fatty acids, triterpenoids, diterpens and glycosides on different cancer cell lines have been shown (40-45). Table 4, shows the GC-MS results that clearly show that *cis* –oleic acid as a fatty acid and *trans-* phytol as a diterpenoid are contents of about 26.91% and 59.87% respectively. On the other hand, the preliminary phytochemical analysis of the extracts showed presence of glycosides and triterpenoids. Therefore, it seems that the potent cytotoxic effects of DCM extract might be related to the presence of these compounds. Whereas some previous reports have indicated a direct relationship between the toxic activity of the BSLT and antiproliferative activity and suggested using BSLT as a low cost and easily mastered technique to replace MTT assay (46-48), some other reports showed indirect correlation (49, 50). Our findings are in consistent with the later. TPC, TFC and antioxidant results are in accordance with previous studies that describe the protective effect of the secondary metabolites (flavonoids and polyphenolic compounds) in diseases that originate from oxidative stress (51-53). Nowadays, researchers have found that the pathophysiology of many diseases such as neurologic disorders, heart diseases and cancer are due to the materials that produce free radicals such as peroxide and lipid peroxyl (54-56). Endogenous antioxidants have been prescribed to inhibit the free radical damage along with consumption of natural exogenous antioxidants that contain an extensive range of polyphenols such as flavonoids and phenolic acids (57-60). According to records in the current literature, the antioxidant ability of MeOH extract, 40% and 60% Sep-pak fractions as well as total phenolic and flavonoid contents were sizeable. In contrast, other extracts and fractions have ignorable activities. Furthermore, phytochemical analysis of MeOH extract revealed presence of alkaloids, tannins, iridoids and flavonoids. Previous researches have reported that the two latter groups of compounds have antioxidant activities (57-59). 

Likewise, there is a positive compatibility and correlation between the free radical scavenging property of test specimens and their total phenolic and flavonoid constituents. A higher donate hydrogen atom ability proportion of 40% and 60% fractions might be due to presence of some polyphenolic compounds, particularly flavonoids that have hydroxyl groups (61, 62). 

For further clarification of the chemical composition of fractions and determination of the relationship between the TPC, TFC and antioxidant activity, 1H-NMR spectra were used. The 1H-NMR spectra of 10% and 20% Sep-pak fractions, showed that they might be rich in glycosilated iridoids. Moreover, remarkable peaks related to Phenolic in 40% fraction. Therefore, phenolics may cause higher values of TPC, TFC and antioxidant activity of 40% fraction. 1H-NMR spectra of 60% fraction, revealed presence of phenylethanoid compounds. Based on the obtained results, it is possible to conclude that 1H-NMR findings are in parallel with TPC and TFC contents as well as antioxidant activity. In a recent study, (Uddine *et al.*) it was reported that extracts with substantial amounts of phenolics, particularly flavonoids had antineoplastic effects (63). Also, researches have reported that the relationship between phenolic and flavonoid ingredients in plant extracts had an anti-cancer effect (64, 65). Although MeOH extract are rich in phenolic compounds, contrary to our expectations, its antiproliferative effect can be easily neglected. However, it is noteworthy that DCM extract of *S. frigida* inhibited proliferation of cancer cells. Findings of this study are in line with those of Valiyari *et al*. (36). 

That study demonstrated that DCM extract of *S. oxysepala* had superior cytotoxic effects compared with MeOH extract on breast cancer cell line. Moreover, results of antioxidant, TPC and TFC tests were in accordance with BSLT data. The result indicated that the potent anti-proliferative effect of DCM extract was not caused by antioxidant and free radical scavenging mechanism and pathway. In general, findings of this present survey imply that the cytotoxic effect of DCM extract of this plant might be effective for treating or decreasing the progress of malignancy of some cancers such as colon and breast cancer. In the current study, no antibacterial activity of extracts vs the selected microorganism strains was observed. However numerous studies were reported the antibacterial activity of some other species of *Scrophularia,* which were found to be rich in phenolic compounds and methanolic extracts (11,15, 66-68). 

## Conclusion

In conclusion the current survey suggests that the existence of some bioactive compounds in DCM and MeOH extracts which inhibit the proliferation of cancerous cells and showed the antioxidant activity with high content of TPC and TFC respectively. It seems that the mechanism of antiproliferative power of this plant was different from the way it was considered for free radical scavenging. 
